# Service-learning outreach to attract high school students to degrees and careers in agricultural sciences

**DOI:** 10.1093/tas/txae012

**Published:** 2024-02-10

**Authors:** Emily A Blanton, Ryan G Anderson, Merritt L Drewery

**Affiliations:** Department of Agricultural Sciences, Texas State University, San Marcos, TX 78666, USA; Department of Agricultural Sciences, Texas State University, San Marcos, TX 78666, USA; Department of Agricultural Sciences, Texas State University, San Marcos, TX 78666, USA

**Keywords:** agricultural workforce, experiential learning, high school students, secondary students, service-learning, USDA

## Abstract

Given projected deficits and a lack of diversity, there is a critical need to recruit and develop the next generation of the agricultural workforce. The objectives of our study were to evaluate if AgCamp, a one day workshop focused on agriculture delivered through a college student-led service-learning platform: (1) increased high school students’ knowledge of agriculture, (2) changed their interests in pursuing degrees and careers in agriculture, and (3) increased their comfort and confidence in communicating with others in agriculture. We hosted high school students at AgCamp and provided them with instruction in animal science, horticulture, and agricultural mechanics. Pre- and post-test survey instruments were developed and distributed at the beginning and end of AgCamp. Data were analyzed with SPSS 26.0 using paired sample *t*-tests. As a result of attending this outreach initiative, high school students (*n* = 26) reported having more knowledge of horticulture (*P* < 0.01) and agricultural mechanics (*P* < 0.01), but not animal science (*P* = 0.12), likely due to greater incoming knowledge of this sub-discipline, as reflected on the pre-test value. High school participants were also more interested in pursuing a college degree (*P* = 0.04) and career (*P* < 0.01) in agriculture and became more confident approaching other high school students (*P* < 0.01), college students (*P* < 0.01), and college faculty (*P* = 0.01) involved in agriculture. Ultimately, participating in AgCamp stimulated high school students’ knowledge and interest in pursuing agricultural degrees and careers, indicating there is value in offering youth outreach as short-term programming to attract students to agriculture.

## Teaser

This research explores the value of a service-learning workshop led by college undergraduates for increasing high school students’ knowledge and interest in pursuing degrees and careers in agricultural sciences.

## Introduction

Employment opportunities for Agricultural and Food Scientists are projected to increase by 8% from 2021 to 2031, which is faster than the average (5%) for all occupations in the United States (Bureau of Labor Statistics, 2023). The most recent United States Department of Agriculture (USDA) Employment Outlook report indicates that, from 2020 to 2025, there will be an annual deficit of 1,300 college graduates to fill requisite science and engineering positions in the food, agriculture, renewable natural resources, and environmental workforce (Fernandez et al., 2019). These projections were made before COVID-19 and, since the pandemic, existing workforce deficits have been exacerbated (Farm Service Agency, 2022).

Beyond deficits in human capital, the agricultural workforce is also challenged by ethnic disparities. Lack of and poor integration of diverse people in the science and engineering workforce impedes creativity and the full potential for innovation (Smith-Doerr et al., 2017). Furthermore, the USDA has historically and recently struggled to engage the agricultural minority workforce (Gonzalez, 2018; Russell et al., 2021) despite the recent adoption of a strategic roadmap for increasing diversity, equity, inclusion, and accessibility in the federal workforce (USDA, 2022).

Hispanic adults hold 9% of ‘Biologists, Agriculturalists, or other Life Scientists’ positions in the United States, whereas Black adults hold only 3% (National Science Foundation, 2019). Of ‘Agricultural Producers’, only 3% are Hispanic and 1% are Black (National Agricultural Statistics Service, 2019). The prevailing underrepresentations likely result from the historical experiences of ethnic minority people in agriculture as poorly compensated laborers working in adverse conditions (Talbert et al., 1999) which may have framed agricultural work (Jean-Philippe et al., 2017) and degrees (Hoover and Scanlon, 1991) in a negative light.

Underrepresentation in agriculture is also reflected in post-secondary study. Ethnic minority students pursue agriculture degrees at lower rates than majority groups (Faulkner et al., 2008). Hispanic and Black students represent only 10% and 3% of Bachelor of Science degree recipients in agriculture disciplines, respectively (National Science Foundation, 2019). Existing literature suggests that ethnic minority students frequently do not receive the knowledge or experiences necessary to develop positive attitudes towards agriculture and, thus, often have a disconnect that prevents them from considering degrees or careers in agriculture (Bowman and Shepard, 1985; Bowen, 1994; Talbert et al., 1999). Feintuch (2009) suggested that minority participation in agriculture should be promoted at the high school level with Drewery et al. (2023) advocating for experiential learning to enhance minority student’s sense of belonging in agriculture.

Beyond ethnicity, younger generations lack interest in agriculture, which contributes to the projected and realized workforce deficits (Holz-Clause and Jost, 1995; Farm Service Agency, 2022). School-based agricultural education students perceive agriculture as solely outdoor labor (Orthel et al., 1989) and would not enroll in agriculture courses to pursue technology, engineering, and/or teaching (Conroy, 2000). Urban youth perceive that most careers in agriculture and natural resources involve a great deal of manual labor and are predominantly associated with crop and animal farming (Jean-Philippe et al., 2017). Although agriculture is a technologically literate and evolving field, youth are often discouraged from entering degree programs or careers by parents, teachers, and mentors due to prevailing perceptions (Jackson and Williams, 2003). This is unfortunate because the agricultural industry consistently innovates and adopts cutting-edge technology (Groher et al., 2020; DeLay et al., 2021), offering careers that emphasize science, technology, engineering, and mathematics (STEM) competencies.

Previous research demonstrates that a week-long workshop improved school-based agricultural education students’ attitudes about food and agricultural sciences (Wiley et al., 1997) and that secondary students who have access to STEM programs have increased interest in pursuing those careers (Kitchen et al., 2018; Hidğe and Aktamiş, 2022). Short-term programming can also have benefits; interacting with an agricultural awareness exhibition increases secondary student’s knowledge about the role of agriculture in their lives (Luckey et al., 2013). There remains a deficit of literature; however, on how service-learning outreach to high school students impacts their knowledge and pursuits related to agriculture. Service-learning is a pedagogical strategy that integrates community service with academic curriculum (Billig, 2000). Service-learning outreach delivered through a college student-led platform could be an effective tool to recruit and engage high school students in agriculture, especially those from ethnic minority groups (Drewery et al., 2023).

The objective of our research was to evaluate if a one day workshop focused on agriculture delivered through a college student-led service-learning platform (1) increased high school students’ knowledge of agriculture, (2) changed their interests in pursuing post-secondary degrees and careers in agriculture, and (3) increased their comfort and confidence in communicating with others in the agricultural community.

## Materials and Methods

These procedures were approved by the Institutional Review Board at Texas State University (#7980); parental consent and minor assent were provided prior to the study. We evaluated the impact of a one day, service-learning workshop (“AgCamp”) on high school students’ perceived knowledge in agriculture; interests in pursuing college degrees and careers in agriculture; and level of confidence and comfort interacting with others in agriculture.

AgCamp was hosted by Texas State University undergraduate students enrolled in a 3000-level course, “Leadership Development in Agricultural Sciences”. Over the course of one semester, these college students invited students from Texas high schools to AgCamp and developed lesson plans and activities to be delivered through service-learning outreach. On the day of AgCamp, college students provided high school students with lectures and demonstrations in animal science, horticulture, and agriculture mechanics; served as near-peer mentors during structured time for informal conversations; and gave facility tours. The impact of developing the service-learning event on the college students has been documented elsewhere (Drewery and Lollar, 2024).

Activities at AgCamp and the intended outcome or value for high school students are outlined in Table 1. While this program was adopted across multiple semesters of AgCamp, the specific lesson or activity may have differed. For example, during one semester, college students who delivered the animal science demonstration may have chosen to lead the high school students in dissecting fetal pigs while, during another semester, college students delivering that same demonstration may have chosen to conduct fecal egg counts with goats.

**Table 1.** Example itinerary for AgCamp, an outreach workshop for high school students delivered by college students through a service-learning platform

Activity	Intended outcome/value for high school students
Introduction/icebreaker and facility tour	Create a comfortable and casual environment for high school students and set expectations for the day
Lesson: animal science	Deliver a lecture-based lesson on Animal Science concepts (e.g., overview of the United States swine industry)
Demonstration: animal science	Involve high school students in a hands-on activity related to Animal Science concepts (e.g., dissecting fetal pigs)
Lesson: horticulture	Deliver a lecture-based lesson on horticulture concepts (e.g., native vs. invasive plants)
Demonstration: horticulture	Involve high school students in a hands-on activity related to horticulture concepts (e.g., succulent propagation)
Lunch and lesson: agricultural careers	Eat lunch and learn about agriculture careers and the United States Department of Agriculture
Lesson: agricultural mechanics	Deliver a lecture-based lesson on agricultural mechanics concepts (e.g., comparison of small- and large-engines)
Demonstration: agricultural mechanics	Involve high school students in a hands-on activity related to agricultural mechanics concepts (e.g., circuit board wiring)
Focus groups	Provide structured time for small groups of high school students to meet with a college student to discuss college and general questions

To analyze the impact of attending AgCamp on high school students, we distributed questionnaire-based survey instruments as a pre-test at the beginning of AgCamp and as a post-test at the end. The instrument included four sections which were: (1) demographic questions (e.g., age, ethnicity, and gender identity; included only on the pre-test), (2) background knowledge of agriculture (i.e., knowledge of the USDA and opportunities in agricultural careers), (3) interests (i.e., interest in attending college, interest in the agriculture field), and (4) confidence and comfort interacting within the agricultural community (i.e., confidence talking to agricultural faculty, comfort level discussing agriculture with peers). Sections two through four of the survey used a five-point Likert scale where one was strongly disagree, three was neutral, and five was strongly agree. Our survey employed questions with a reverse scale and negative wording such as, “I feel graduating from college is not possible for me.” Responses were reverse-scored prior to analysis.

Before distributing the survey, we consulted a panel of content area experts (*n* = 11) to establish face, construct, and criterion validity of the instrument. These experts were Texas State University faculty with survey development and service-learning expertise. The instrument was revised until approved by the entire panel.

We distributed the survey to high school students attending AgCamp in the fall 2021, spring 2022, and fall 2022 semesters. We used data collected from the 11 Texas high school students who participated in AgCamp during the fall 2021 semester to establish reliability of our instrument. Cronbach’s alpha of the instrument was *α* = 0.76, which we interpreted as acceptable reliability (George and Mallery, 2003). Our final dataset included data from spring and fall 2022, but not fall 2021.

In spring and fall 2022, we received 28 responses to our pretest survey and 29 responses to our post-test survey. Data were cleaned for participants who completed both surveys and answered at least 75% of the questions on both; those who did not meet these criteria (*n* = 3) were removed from the dataset for a final convenience sample of 26.

We analyzed pre- and post-test data with SPSS 26.0 using paired sample *t*-tests and measures of central tendency. One-way ANOVA was also used to determine if there were differences in how ethnic minority vs. majority students responded to the pretest. For this, Black and Hispanic students were grouped into an independent variable, “ethnic minority” as implemented by Drewery et al. (2023). If significant (*P* ≤ 0.05) differences for ethnic minority vs. majority were observed for the pretest, the corresponding post-test response was also analyzed via one-way ANOVA to determine if pretest differences persisted into the post-test.

## Results

Participants were predominately male (58%); and were Hispanic or Latino (62%), White or Caucasian (31%), Black or African American (4%), and other or not listed (4%; Table 2). The grade classification of respondents was freshmen (15%, *n* = 4), sophomores (8%, *n* = 2), juniors (12, *n* = 3), and seniors (65%, *n* = 17) in high school. Participant ages ranged from 14 to 18 years with one participant preferring not to answer.

**Table 2.** Demographics of high school students (*n* = 26) who participated in AgCamp

Gender	%
Male	57.7
Female	38.5
Prefer not to answer	3.8
Ethnicity	%
White or Caucasian	30.8
Black or African American	3.8
Hispanic or Latino	61.5
Other or not listed	3.9
Level of study	%
Freshman	15
Sophomore	8
Junior	12
Senior	65
Age, years	%
14	15.4
15	3.8
16	15.4
17	34.6
18	26.9
Prefer not to answer	3.8

Participation in AgCamp significantly increased (*P* < 0.01) high school students’ knowledge of the available career opportunities in the agricultural sciences sector (Table 3) from a pre-test mean of 3.50 (*SEM* = 0.71) to a post-test mean of 4.23 (*SEM* = 0.59). High school students also better understood who the USDA was as a result of AgCamp; there was a significant increase (*P* < 0.01) from a pre-test mean of 3.38 (*SEM* = 0.90) to a post-test mean of 4.04 (*SEM* = 0.60). Furthermore, high school students significantly increased (*P* < 0.01) their understanding of what employment opportunities exist within the USDA from a pre-test mean of 2.81 (*SEM* = 0.63) to a post-test mean of 3.88 (*SEM* = 0.77).

**Table 3.** Change in high school students’(*n* = 26) perceptions of their content area knowledge as a result of participating in AgCamp

Prompt	Pre-test	Post-test	*P*-value
I understand what career opportunities are available in agriculture science sectors.	3.50 (0.71)	4.23 (0.59)	<0.01
I understand who the United States Department of Agriculture (USDA) is.	3.38 (0.90)	4.04 (0.60)	<0.01
I understand what employment opportunities exist within the USDA federal agency.	2.81 (0.63)	3.88 (0.77)	<0.01
I feel I have a basic knowledge of agricultural mechanics.	2.90 (0.89)	3.81 (0.63)	<0.01
I feel I have a basic knowledge of horticulture.	2.88 (0.95)	3.84 (0.83)	<0.01
I feel I have a basic knowledge of animal science.	3.50 (0.81)	3.80 (0.80)	0.12

Data are presented: Mean (SEM).

The agricultural sub-disciplines included in AgCamp were animal science, horticulture, and agricultural mechanics, with dedicated time for a lesson and demonstration in each. We asked high school participants about their “basic knowledge” of each sub-discipline. Due to their participation in AgCamp, high school students perceived that their basic knowledge of agricultural mechanics and horticulture increased significantly (*P* < 0.01) with pre-test means of 2.90 (*SEM* = 0.89) and 2.88 (*SEM* = 0.95), respectively, and post-test means of 3.81 (*SEM* = 0.63) and 3.84 (*SEM* = 0.83), respectively. There was not a significant change (*P* = 0.12) in high school students’ perceptions of their basic knowledge of animal science as a result of AgCamp; the pre-test mean was 3.50 (*SEM* = 0.81) and post-test mean was 3.80 (*SEM* = 0.80).

In determining high school students’ post-secondary degree and career interests before and after AgCamp, we observed a significant increase (*P* = 0.04) in their interest in pursuing a college degree in agricultural sciences with a pre-test mean of 3.27 (*SEM* = 0.87) and a post-test mean of 3.65 (*SEM* = 0.85; Table 4). We also observed a significant increase (*P* < 0.01) in high school students’ interest in pursuing a career in agriculture with a pre-test mean of 3.58 (*SEM* = 0.86) and post-test mean of 3.96 (*SEM* = 0.87). High school participants’ pre- and post-test means were the same (3.54) regarding their interest in pursuing a college degree, but not in agricultural sciences (*P* = 1.00). Finally, their interest in pursuing a degree at Texas State University, the host institution, significantly increased (*P* < 0.01) from a pre-test mean of 3.27 (*SEM* = 0.78) to a post-test mean of 3.77 (*SEM* = 0.71).

**Table 4.** Change in high school students’(*n* = 26) post-secondary degree and career interests as a result of participating in AgCamp

Prompt	Pre-test^1^	Post-test	*P*-value
I am interested in having a career in the agriculture industry.	3.58 (0.86)	3.96 (0.87)	<0.01
I am interested in pursuing a college degree in agricultural sciences.	3.27 (0.87)	3.65 (0.85)	0.04
I am interested in pursuing a college degree, but not in agricultural sciences.	3.54 (0.76)	3.54 (1.10)	1.00
I am interested in pursuing a college degree at Texas State University.	3.27 (0.78)	3.77 (0.71)	<0.01

^1^Data are presented: Mean (SEM).

High school students’ comfort level in having conversations with other high schoolers involved in agriculture significantly increased (*P* < 0.01) from a pretest mean of 3.36 (*SEM* = 0.81) to a post-test mean of 4.00 (*SEM* = 0.65; Table 5). There was also an increase (*P* < 0.01) in their confidence in approaching other high school students involved in agriculture, from a pre-test mean of 3.36 (*SEM* = 0.76) to a post-test mean of 4.04 (*SEM* = 0.73).

**Table 5.** Change in high school students’ (*n* = 26) level of comfort and confidence interacting with others in agriculture as a result of participating in AgCamp

Prompt	Pre-test^1^	Post-test	*P*-value
I feel comfortable having conversations and discussions about agriculture with other high school students involved in agriculture.	3.36 (0.81)	4.00 (0.65)	<0.01
I feel confident approaching other high school students involved in agriculture.	3.36 (0.76)	4.04 (0.73)	<0.01
I feel comfortable having conversations and discussions about agriculture with college students.	3.40 (0.87)	4.20 (0.82)	<0.01
I feel confident approaching college students involved in agriculture.	3.44 (0.92)	4.08 (0.76)	<0.01
I feel comfortable having conversations and discussions about agriculture with college professors.	3.36 (0.95)	3.88 (0.73)	0.01
I feel confident approaching college professors involved in agriculture.	3.40 (1.00)	4.00 (0.87)	0.01
I feel comfortable having conversations and discussions about agriculture with professionals employed in the agricultural sciences sector.	3.40 (0.96)	3.96 (0.84)	0.01
I feel confident approaching professionals employed in the agriculture sciences sector.	3.56 (1.08)	3.92 (0.76)	0.14

^1^Data are presented: Mean (SEM)

When analyzing if ethnic minority vs. majority impacted pretest responses, there was a significant effect on high school students’ response to “I feel confident approaching other high school students involved in agriculture” such that ethnic minority students (i.e., Hispanic or Black) had a lower pre-test mean (*P* = 0.05) than majority (i.e., White) students, 3.06 (*SEM* = 0.75) vs. 3.75 (*SEM* = 0.71), respectively. However, this difference did not persist for the post-test (*P* = 0.16) with ethnic minority students having a post-test mean of 3.94 (*SEM* = 0.77) and majority students having a post-test mean of 4.38 (*SEM* = 0.52).

High school students felt significantly (*P* < 0.01) more comfortable having conversations and discussions about agriculture with college students as a result of AgCamp; the pre-test mean was 3.40 (*SEM* = 0.87) and post-test mean was 4.20 (*SEM* = 0.82; Table 5). High school students also became significantly more confident (*P* < 0.01) approaching college students involved in agriculture, from a pre-test mean of 3.44 (*SEM* = 0.92) to a post-test mean of 4.08 (*SEM* = 0.76).

High school students felt significantly (*P* = 0.01) more comfortable having conversations about agriculture with college professors (Table 5); the pre-test mean was 3.36 (*SEM* = 0.95) and post-test mean was 3.88 (*SEM* = 0.73). They also became more confident in approaching these professors (*P* = 0.01), from a pre-test mean of 3.40 (*SEM* = 1.00) to a post-test mean of 4.00 (*SEM* = 0.87). There was a positive change in high school students’ level of comfort (*P* = 0.01), but not confidence (*P* = 0.14), in interacting with professionals employed in the agriculture sector.

## Discussion

We evaluated if AgCamp, a service-learning outreach workshop led by college undergraduates, impacted high school student’s knowledge and pursuits related to agriculture by distributing pre- and post-test surveys to high school attendees across two semesters.

Pre-test means demonstrate that our high school participants felt they had more basic knowledge about animal science than horticulture or agriculture mechanics. Within colleges of agriculture, Animal Science is often the most frequently reported major with greater enrollment of students who are also involved in agriculture clubs or activities (Barkley and Parrish, 2005). Further, a survey conducted on Animal Science majors at a land-grant institution indicated that 54% to 65% were members of co-curricular agriculture organizations in high school (Winkel et al., 2020). We did not ask participants about their involvement in agriculture organizations, but it is likely that their co-curricular experiences drove the self-reported increase in animal science knowledge on the pretest.

There was an increase in high school students’ perceptions of their knowledge of horticulture and agricultural mechanics, but not animal science, due to AgCamp. On the post-test, high school participants reported having nearly equal knowledge of each sub-discipline, indicating that AgCamp rounded out their knowledge of different sub-fields under the broad umbrella of agriculture. This highlights an opportunity for post-secondary programs to provide targeted interventions for high school students that educate them about horticulture and agricultural mechanics while also exciting them about opportunities in those areas. These initiatives have value as horticulture and other agriculture, non-animal science majors have recently struggled recruiting and retaining students (Dole, 2015) and there are concerns that horticulture is becoming a lost major and career path (Pritts and Park, 2013). Previous research agrees with ours where service-learning workshops increased high school students’ subject-matter interest and positively affected their attitudes about pursuing degrees within a given discipline (Morgan and Streb, 2003; Fraze et al., 2011).

Holz-Clause and Jost (1995) reported that youth in the United States are apathetic towards agricultural careers, underlining the need to excite them about related opportunities. Our results demonstrate that high school students had increased interest in pursuing agriculture degrees and careers after attending AgCamp. As our sample was majority-minority (i.e., 65% identified as Hispanic or Black), AgCamp increased the interest of historically underrepresented students in agriculture. While promising, these findings are limited due to sample size (*n* = 26) and, because participants were not tracked longitudinally, we cannot determine if the reported shift in interests became actionable. Research into short-term programming that develops a recruiting “pipeline” from secondary to post-secondary study should ideally follow up with participants after the intervention to assess long-term outcomes.

Okerson (2016) reported that the intangible feeling of comfort on campus contributes to high school students’ decisions when picking a college. Providing high school students with opportunities to interact with college students shapes their view of themselves in college (Hooker and Brand, 2010; Swanson et al., 2021). We designated time at AgCamp for high school students to interact with the university community and tour facilities, which likely drove the increased interest in attending the host university as indicated on the post-test. Our findings indicate that service-learning workshops led by college undergraduates may be an effective recruiting mechanism for post-secondary institutions.

Esters and Bowen (2005) demonstrated that educational experiences during high school influence future career choices. In our study, high school students were more interested in pursuing opportunities within agriculture after attending AgCamp. However, as college enrollment decisions may be influenced at or before junior high (Hoover and Scanlon, 1991; Hidğe and Aktamiş, 2022), we suggest similar research that targets elementary or junior high audiences to provide insight into the ideal level of study for future youth outreach.

Informed by our findings, we recommend that service-learning outreach should be offered to recruit high school students for degrees and careers in agriculture. This outreach should be led by college students and provide structured and non-structured time for interactions between high school students, college students, faculty, and industry representatives. Youth outreach such as the initiative described here is especially important in regions with significant ethnic minority representation and fields with workforce deficits.

## Supplementary Material

txae012_suppl_Supplementary_Material
