# Supplementary material for: Service-learning outreach to attract high school students to degrees and careers in agricultural sciences
Source: Transl Anim Sci. 2024 Feb 10;8:txae012. doi: 10.1093/tas/txae012 (PMC10858439; doi:10.1093/tas/txae012)
Supplement: txae012_suppl_Supplementary_Material [file txae012_suppl_supplementary_material.docx]

Impact of service-learning on secondary students (pre-test)

Start of Block: Section 1: Demographics

What is your name?

________________________________________________________________

What is your gender identity?

- Male
- Female
- Non-binary
- Prefer not to say

What is your age, in years? Please enter whole numbers only.

________________________________________________________________

What is your high school classification?

- Freshman
- Sophomore
- Junior
- Senior

Which of the following would best describe you?

- American Indian or Alaskan Native
- Asian, Native Hawaiian, or Other Pacific Islander
- Black or African American
- White or Caucasian
- Hispanic or Latino
- A race or ethnicity not listed here

End of Block: Section 1: Demographics

Start of Block: Section 2: Background Knowledge/Interests

I understand what career opportunities are available in the agricultural sciences sector.

- Strongly disagree
- Disagree
- Neutral
- Agree
- Strongly agree

I understand who the United States Department of Agriculture (USDA) is.

- Strongly disagree
- Disagree
- Neutral
- Agree
- Strongly agree

I understand what employment opportunities exist within the USDA federal agency.

- Strongly disagree
- Disagree
- Neutral
- Agree
- Strongly agree

I am interested in having a career in the agricultural industry.

- Strongly disagree
- Disagree
- Neutral
- Agree
- Strongly agree

I am interested in pursuing a college degree in Agricultural Sciences.

- Strongly disagree
- Disagree
- Neutral
- Agree
- Strongly agree

I am interested in pursuing a college degree, but not in Agricultural Sciences.

- Strongly disagree
- Disagree
- Neutral
- Agree
- Strongly agree

I am interested in pursuing a college degree at Texas State University.

- Strongly disagree
- Disagree
- Neutral
- Agree
- Strongly agree

I feel attending college is not possible for me.

- Strongly disagree
- Disagree
- Neutral
- Agree
- Strongly agree

I feel graduating from college is not possible for me.

- Strongly disagree
- Disagree
- Neutral
- Agree
- Strongly agree

I am confident that I will eventually be employed in a field of my choice.

- Strongly disagree
- Disagree
- Neutral
- Agree
- Strongly agree

I do not know how to achieve my professional ambitions or goals.

- Strongly disagree
- DIsagree
- Neutral
- Agree
- Strongly agree

I feel AgCamp will help me understand the career pathway I want to pursue.

- Strongly disagree
- Disagree
- Neutral
- Agree
- Strongly agree

I feel AgCamp will help me understand which subject area in agriculture I am genuinely interested in.

- Strongly disagree
- DIsagree
- Neutral
- Agree
- Strongly agree

I feel AgCamp will help me understand which subject area in agriculture I would be willing to learn more about.

- Strongly disagree
- Disagree
- Neutral
- Agree
- Strongly agree

I feel I have a basic knowledge of Animal Science.

- Strongly disagree
- DIsagree
- Neutral
- Agree
- Strongly agree

I feel I have a basic knowledge of Agricultural Mechanics.

- Strongly disagree
- DIsagree
- Neutral
- Agree
- Strongly agree

I feel I have a basic knowledge of Horticulture.

- Strongly disagree
- DIsagree
- Neutral
- Agree
- Strongly agree

I feel comfortable having conversations and discussions about agriculture with other high school students involved in agriculture.

- Strongly disagree
- Disagree
- Neutral
- Agree
- Strongly agree

I feel confident approaching other high school students involved in agriculture.

- Strongly disagree
- Disagree
- Neutral
- Agree
- Strongly agree

I feel comfortable having conversations and discussions about agriculture with college students.

- Strongly disagree
- Disagree
- Neutral
- Agree
- Strongly agree
- Non-applicable

I feel confident approaching college students involved in agriculture.

- Strongly disagree
- Disagree
- Neutral
- Agree
- Strongly agree
- Non-applicable

I feel comfortable having conversations and discussions about agriculture with college professors.

- Strongly disagree
- Disagree
- Neutral
- Agree
- Strongly agree
- Non-applicable

I feel confident approaching college professors involved in agriculture.

- Strongly disagree
- Disagree
- Neutral
- Agree
- Strongly agree
- Non-applicable

I feel comfortable having conversations and discussions about agriculture with professionals employed in the agricultural sciences sector.

- Strongly disagree
- Disagree
- Neutral
- Agree
- Strongly agree
- Non-appplicable

I feel confident approaching professionals employed in the agricultural sciences sector.

- Strongly disagree
- Disagree
- Neutral
- Agree
- Strongly agree
- Non-appplicable

I typically ask questions when I do not understand something during a lesson.

- Strongly disagree
- Disagree
- Neutral
- Agree
- Strongly agree

I typically contribute to class discussions during lessons.

- Strongly disagree
- Disagree
- Neutral
- Agree
- Strongly agree

I feel I learn better with hands-on activities during lessons or demonstrations.

- Strongly disagree
- DIsagree
- Neutral
- Agree
- Strongly agree

End of Block: Section 2: Background Knowledge/Interests

Impact of service-learning on secondary students (post-test)

Start of Block: Section 1: Demographics

What is your name?

________________________________________________________________

End of Block: Section 1: Demographics

Start of Block: Section 2: Background Knowledge/Interests

I understand what career opportunities are available in the agricultural sciences sector.

- Strongly disagree
- Disagree
- Neutral
- Agree
- Strongly agree

I understand who the United States Department of Agriculture (USDA) is.

- Strongly disagree
- Disagree
- Neutral
- Agree
- Strongly agree

I understand what employment opportunities exist within the USDA federal agency.

- Strongly disagree
- Disagree
- Neutral
- Agree
- Strongly agree

I am interested in having a career in the agricultural industry

- Strongly disagree
- Disagree
- Neutral
- Agree
- Strongly agree

I am interested in pursuing a college degree in Agricultural Sciences.

- Strongly disagree
- Disagree
- Neutral
- Agree
- Strongly agree

I am interested in pursuing a college degree, but not in Agricultural Sciences.

- Strongly disagree
- Disagree
- Neutral
- Agree
- Strongly agree

I am interested in pursuing a college degree at Texas State University.

- Strongly disagree
- Disagree
- Neutral
- Agree
- Strongly agree

I feel attending college is not possible for me.

- Strongly disagree
- Disagree
- Neutral
- Agree
- Strongly agree

I feel graduating from college is not possible for me.

- Strongly disagree
- Disagree
- Neutral
- Agree
- Strongly agree

I am confident that I will eventually be employed in a career path of my choice.

- Strongly disagree
- Disagree
- Neutral
- Agree
- Strongly agree

I do not know how to achieve my professional ambitions or goals.

- Strongly disagree
- Disagree
- Neutral
- Agree
- Strongly agree

I feel AgCamp has helped me understand the career pathway I want to pursue.

- Strongly disagree
- Disagree
- Neutral
- Agree
- Strongly agree

I feel AgCamp has helped me understand which subject area in agriculture I am genuinely interested in.

- Strongly disagree
- Disagree
- Neutral
- Agree
- Strongly agree

I feel AgCamp has helped me understand which subject area in agriculture I would be willing to learn more about.

- Strongly disagree
- Disagree
- Neutral
- Agree
- Strongly agree

I feel I have a basic knowledge of Animal Science.

- Strongly disagree
- Disagree
- Neutral
- Agree
- Strongly agree

I feel I have a basic knowledge of Agricultural Mechanics.

- Strongly disagree
- Disagree
- Neutral
- Agree
- Strongly agree

I feel I have a basic knowledge of Horticulture.

- Strongly disagree
- Disagree
- Neutral
- Agree
- Strongly agree

End of Block: Section 2: Background Knowledge/Interests

Start of Block: Section 3: Learning Outcomes

I feel comfortable having conversations and discussions about agriculture with other high school students involved in agriculture.

- Strongly disagree
- Disagree
- Neutral
- Agree
- Strongly agree
- Non-applicable

I feel confident approaching other high school students involved in agriculture.

- Strongly disagree
- Disagree
- Neutral
- Agree
- Strongly agree
- Non-applicable

I feel comfortable having conversations and discussions about agriculture with college students.

- Strongly disagree
- Disagree
- Neutral
- Agree
- Strongly agree
- Non-applicable

I feel confident approaching college students involved in agriculture.

- Strongly disagree
- Disagree
- Neutral
- Agree
- Strongly agree
- Non-applicable

I feel comfortable having conversations and discussions about agriculture with college professors.

- Strongly disagree
- Disagree
- Neutral
- Agree
- Strongly agree
- Non-applicable

I feel confident approaching college professors involved in agriculture.

- Strongly disagree
- Disagree
- Neutral
- Agree
- Strongly agree
- Non-applicable

I feel comfortable having conversations and discussions about agriculture with professionals employed in the agricultural sciences sector.

- Strongly disagree
- Disagree
- Neutral
- Agree
- Strongly agree
- Non-applicable

I feel confident approaching professionals employed in the agriculture sciences sector.

- Strongly disagree
- Disagree
- Neutral
- Agree
- Strongly agree
- Non-applicable

I asked questions when I did not understand something during today's lessons.

- Strongly disagree
- Disagree
- Neutral
- Agree
- Strongly agree

I contributed to class discussions during today's lessons.

- Strongly disagree
- Disagree
- Neutral
- Agree
- Strongly agree

I feel I learned better with hands-on activities during today's lessons in agriculture mechanics, horticulture, and animal science.

- Strongly disagree
- Disagree
- Neutral
- Agree
- Strongly agree

Did you find the agricultural mechanic's lesson engaging?

- Strongly disagree
- Disagree
- Neutral
- Agree
- Strongly agree

Did you find the agricultural mechanic's demonstration engaging?

- Strongly disagree
- Disagree
- Neutral
- Agree
- Strongly agree

Did you find the horticulture lesson engaging?

- Strongly disagree
- Disagree
- Neutral
- Agree
- Strongly agree

Did you find the horticulture demonstration engaging?

- Strongly disagree
- Disagree
- Neutral
- Agree
- Strongly agree

Did you find the animal science lesson engaging?

- Strongly disagree
- Disagree
- Neutral
- Agree
- Strongly agree

Did you find the animal science demonstration engaging?

- Strongly disagree
- Disagree
- Neutral
- Agree
- Strongly agree

End of Block: Section 3: Learning Outcomes

Start of Block: Block 4: Suggestions and Overall Experience

I would encourage and recommend other high school students to attend AgCamp.

- Strongly disagree
- Disagree
- Neutral
- Agree
- Strongly agree

If I were given a chance, I would attend AgCamp again.

- Strongly disagree
- Disagree
- Neutral
- Agree
- Strongly agree

How would you rate your overall experience at AgCamp.

- Very poor
- Poor
- Average
- Good
- Excellent

What could we have included or changed to improve your overall experience at AgCamp?

________________________________________________________________

End of Block: Block 4: Suggestions and Overall Experience
